# *Bacillus thuringiensis*: A Broader View of Its Biocidal Activity

**DOI:** 10.3390/toxins16030162

**Published:** 2024-03-20

**Authors:** Leopoldo Palma, Diego Herman Sauka, Jorge E. Ibarra

**Affiliations:** 1 Instituto de Biotecnología y Biomedicina (BIOTECMED), Universitat de València, 46100 Burjassot, Spain; 2 Consejo Nacional de Investigaciones Científicas y Técnicas (CONICET), Ciudad Autónoma de Buenos Aires 1425, Argentina; 3 Instituto de Microbiología y Zoología Agrícola (IMYZA), Instituto Nacional de Tecnología Agropecuaria (INTA), Hurlingham, Ciudad Autónoma de Buenos Aires 1686, Argentina; 4 Departamentos de Biotecnología y Bioquímica, Centro de Investigación y de Estudios Avanzados del IPN-Unidad Irapuato, Apartado Postal 629, Irapuato 36500, Guanajuato, Mexico

*Bacillus thuringiensis* (Bt) is a Gram-positive bacterium that forms spores and produces parasporal crystalline inclusions containing Cry and Cyt proteins [1]. These proteins exhibit toxicity against various insect orders, nematodes, and human cancer cells [2,3]. Widely utilized as bioinsecticides, Bt strains and their insecticidal proteins effectively control caterpillars, beetles, flies, mosquitoes, and blackflies. During vegetative growth, Bt can also secrete insecticidal proteins targeting lepidopterans (Vip3) and coleopterans (Vpab1/Vpab2). Another less-explored secretory protein, Mpp5Aa1 (formerly Sip1A), has also been described to exhibit activity against coleopteran pests [4]. These features have bestowed Bt as the most specific and effective tool for the control of insect pests for several years, either through insecticidal formulations (a mix of spore and parasporal crystals) or by the production of insecticidal proteins in transgenic plants (Bt plants) [5]. However, some species, such as *Plutella xylostella* (Lepidoptera), have developed field resistance to both formulated products and insecticidal proteins expressed in transgenic plants [2], making screenings for novel strains and pesticidal proteins highly essential in order to provide novel tools for the control of pests and the management of insect resistance. 

The aim of this Special Issue, “*Bacillus thuringiensis*: A Broader View of Its Biocidal Activity”, was to gather information on novel Bt strains and proteins showing novel pesticidal properties to provide biotechnological tools with useful resources for pest control in agriculture and to incentivize researchers to perform such necessary research. This subject has been of great interest, allowing the publication of 12 research papers from top researchers working in the field worldwide, which have shed light on the diverse and multifunctional properties of novel (unreported) Bt strains and proteins. Beyond the conventional focus, these studies delve into various aspects, including structural insights, insecticidal proteins, toxin interactions, and the evaluation of novel strains.

Additionally, this editorial aims to provide an overview of key findings from these papers; for example, Unzue et al. showcased the broad spectrum of Bt applications and their potential implications, such as the multifunctional properties of Bt strain BST-122, encompassing the biocidal properties beyond those of the well-known pesticidal parasporal crystals (contribution 1). Li et al. presented a deeper understanding of the structure of Cry5B, unraveling the active form of Cry5B that could contribute to the development of more effective and targeted nematicidal products (contribution 2). The study by Best et al. showed the crystal structure of Bt Tpp80Aa1 protein (formerly Cry80Aa1) and its interaction with galactose-containing glycolipids, unveiling molecular details that may have implications for understanding the specificity and selectivity of Bt toxins (contribution 3). Two papers about binary Mpp23Aa/Xpp37Aa proteins and the novel Bt strain Bt_UNVM-84 were presented by de Oliveira et al. and Sauka et al., respectively, highlighting the potential application of these resources in pest control, particularly against *Anthonomus grandis* (Coleoptera), a harmful pest causing high economic losses in the cotton industry in the Americas (contributions 4 and 10). Covering proteins with activity against mosquitoes, Lai et al.’s paper examines the role of Cyt proteins in enhancing the activity of other Bt toxins against *Aedes albopictus*, emphasizing the cooperative interactions between different toxin classes. Understanding these synergies could contribute to the development of more effective mosquito control strategies (contribution 5). Yang’s study described a Cry protein with activity against the rice leaffolder *Cnaphalocrocis medinalis*. This paper explored the processing properties and potency of Cry toxins in the context of rice leaffolder control. Insights into the interaction between a Cry protein and this target pest are crucial for optimizing their efficacy in agricultural settings (contribution 6). The study by Xue et al. covered novel synergistic interactions beyond those known among different insecticidal proteins. In this paper, a new synergistic interaction between the extracellular polysaccharide from Bt subsp. *kurstaki* HD-270 and the insecticidal protein Cry1Ac is described, examining this particular synergistic activity and providing valuable information on the interplay between different components in Bt formulations (contribution 7). Trisyono et al. presented a paper covering the baseline susceptibility of field populations of *Ostrinia furnacalis* in Indonesia to Cry1A.105 events and Cry2Ab2 proteins were also covered, assessing the susceptibility of this pest to specific Cry proteins. This research lays the groundwork for understanding the dynamics of resistance and informs us about the sustainable use of Bt technologies in agriculture (contribution 8). Interesting outputs about the field evaluation of transgenic cotton expressing Mpp51Aa2 (formerly Cry51Aa2) as a management tool for the cotton fleahopper *Pseudatomoscelis seriatus* were also published in this Special Issue, contributing valuable data for its implementation. Finally, two papers presented by Hou et al. and Shao et al. describe interesting aspects related to the yet poorly understood mode of action of Vip3A proteins, focusing on molecular details of Vip3A proteins and highlighting specific domains involved in receptor binding and liposomal membrane disruption, respectively (contributions 11 and 12).

In conclusion, the collection of papers featured in this editorial underscores the versatility and complexity of Bt as a bioinsecticide. From structural insights to practical field applications, researchers continue to unravel the potential of Bt strains in addressing agricultural and public health challenges. These findings collectively contribute to the ongoing efforts to harness the full potential of Bt-based technologies for sustainable pest management.
